# Institutional Notes and News

**Published:** 1909-10-30

**Authors:** 


### Institutional Notes and News.

### THE NORFOLK AND NORWICH HOSPITAL.

Foundation Stone Laid by The King.

On Monday afternoon last His Majesty the King laid the foundation stone of the new isolation block of the Norfolk and Norwich Hospital. His Majesty was received by the Earl of Leicester (President), and the Bishop of Thetford. The following gentlemen had the honour of being presented to the King: Mr. Walter E. Hansell, the Chairman of the Board of Management (the Rev. Harvey W. G. Thursby), the Vice-Chairman of the Board (the Ven. Archdeacon Sidney Pelham), the Consulting Physician (Sir Peter Eade, M.D.), the Consulting Surgeons (Dr. Michael Beverley and Mr. H. S. Robinson), the Senior Physician (Dr. Samuel J. Barton), the Senior Surgeon (Mr. Samuel H. Burton), the Chairman of the Building Committee (Mr. Eustace Gurney), the Architect (Mr. E. T. Boardman), and the Lady Superintendent (Miss Florence A. Cann).

The King having taken his place on the platform erected for the occasion, the Bishop of Norwich offered prayer, after which Lord Leicester presented an address, to which His Majesty in reply said :—

I thank you for your loyal address of welcome, which your lordship has presented to me from the governing body and friends of the Norfolk and Norwich Hospital. I well remember my visits to you in 1872 and 1884. The hospital is, and always will be, associated in my mind with the personality of the late Lord Leicester, a man whom, as you know, I held in the highest esteem and regard during the many years of my residence in your county. It was of great interest to me to hear the account of the good work performed by the hospital since its opening. I am glad to say that a want long felt by the hospital authorities, and one which has in many cases hampered their efforts, has now been supplied, and it is a source of gratification to me to open to-day the extension of the hospital designed as an isolation block for the reception of such cases as cannot without risk of infection be allowed to associate with other patients. No greater blessings exist for the poor classes of this country than the institutions which provide for their relief and care in illness, and any movement to establish new buildings for the purpose of extending those already in existence, or to render them more efficient, will always have my warmest support. I am sure that those charitable men and women who have liberally contributed to your hospital in the past will themselves continue their support, and will by their efforts stimulate others to assist this noble work of charity. The encouragement you give to your nurses to join the Nursing Service of the Territorial Forces meets with my utmost cordial approval. In matters of life and death the services of the trained nurse are no less essential than those of the physician or surgeon. I pray that the blessing of God may rest upon your labours.

His Majesty then laid the foundation-stone of the new extension of the hospital, and finally left by motor-car for Crown Point.

### CRANLEIGH COTTAGE HOSPITAL.

On Friday, October 1, the Cranleigh Village Hospital, the first cottage hospital to be established in England, reached its jubilee, having been opened on October 1, 1859. The event was unmarked by any special celebration, but the hospital was gaily decorated with flags during the day. The original hospital was in the old rectory, a small old-world cottage, admirably suited for the purpose. For many years Dr. Napier, its original founder, presided over its destinies. In 1859 there were two small wards, and the number of patients treated at the hospital was about 21. Year by year, however, the hospital has grown in usefulness, and to-day the average number of patients under treatment each year is between 60 and 70. The scope of the hospital has been gradually extended, and it now caters for a very large district, including the parishes of Cranleigh, Ewhurst, Dunsfold, Hascombe, Alfold, Rudgwick, Peaslake, and Holmbury St. Mary. Some seven or eight years ago an important and much needed enlargement was carried out. Two new wards, each with four beds, lavatories, and bathroom, were built at the rear of the old cottage, to which they are connected by a vestibule. One of the wards was the gift of Mr. P. Ralli, as a memorial to his wife, and the other was provided by private subscriptions. The cottage is now used as the nurses' home. Quite recently the committee, to mark the jubilee of the hospital, have, at their own expense, had the cottage repainted, and brought out the fine oak beams in the rooms, thus restoring the building to its ancient state, making it quite a show place.

### RAMSGATE'S NEW HOSPITAL.

Ramsgate has at last become possessed of a new hospital, and it certainly was not before a new institution of that description was needed. The old building was far too small, and far too cramped for room to accommodate the number of cases, both accidental and general, that occur in a town the size of Ramsgate. Therefore, it is with no feelings of regret that the old building is vacated, and the new one inhabited. The hospital is up to date in every possible way, and the staff will be able to treat both medical and surgical cases with all the latest appliances.

### DUNDEE INFIRMARY.

The income for the year, according to the recently issued annual report, amounted to £14,292 12s. 2d., an increase over last year of £1,674 18s. 1d., arising mainly from several large donations applicable to current expenses, and increased receipts for board for patients. Ordinary expenditure amounted to £15,682 18s. 6d., being £102 4s. less than last year. Extraordinary expenditure for completion of the structural alterations which have been in progress for several years amounted to £2,786 18s. 6d., which, added to the ordinary expenditure, making a total of £18,469 17s., resulted in an excess of expenditure over income of £4,177 4s. 10d. The directors record their sense of the great loss which they sustained through the death of Mr. David Gordon Stewart, who for more than 40 years faithfully and efficiently discharged the duties of Secretary and Law Agent of the Corporation. The soundness of his judgment and his knowledge of detail gave great weight to the opinions he expressed, while at all times he retained the confidence and respect of those whom he was called upon to advise. To succeed Mr. Stewart, the directors have appointed as Law Agent Mr. Henry A. Pattullo, and as Secretary Mr. George B. Brough, who for many years was assistant to Mr. Stewart. Twelve months ago the directors for the second time granted the application of the Town Council to accommodate the municipal dispensary for the treatment of pulmonary tuberculosis, and to supply the patients with the medicines prescribed by the medical officer. The dispensary has been very successful. The number of new patients attending was practically the same as during last year. The number of attendances exceeded 2,000. The directors, accordingly, have acceded to the request for a continuance of the privileges accorded to the town authorities.

### THE STOKE GUARDIANS AND NORTH STAFFORDSHIRE INFIRMARY.

A meeting of the Stoke-on-Trent Board of Guardians was held on Wednesday, the 13th inst., at which notice of motion was given to increase the guardians' subscription to such an amount as would entitle them to be represented on the management committee of the North Staffordshire Infirmary. The annual subscription of the guardians in the past has been two guineas.

### NORFOLK AND NORWICH EYE INFIRMARY.

The scheme for the amalgamation of the Institution with the Norfolk and Norwich Hospital has been submitted to every subscriber. A special general meeting of subscribers was summoned and held on Friday, October 16, 1908, at which a resolution agreeing to the same was (subject to sanction of Charity Commissioners), passed without dissentient. A feature of the year's work, particularly of the latter portion of it, has been the number of school children attending on account of errors of refraction. Such errors usually require for their efficient treatment by glasses attendance of the patient at the hospital on three separate occasions. It is, however, gratifying to note that in the case referred to, the parents do bring their children for special treatment by medical men.

### LABOUR AND THE NORTH STAFFORDSHIRE INFIRMARY.

At a meeting of the North Staffordshire Trades and Labour Council at the Town Hall, Hanley, on Wednesday, October 13, the following resolution was passed :—" That this Council is of the opinion that the articles of Sir Henry Burdett relating to the North Staffordshire Infirmary, which have been published in the *Sentinel* have done a great service to the people of North Staffordshire, and the Council feels that it is essential that Labour should be adequately represented on the governing body of that institution and its various committees. Further, that a committee of the Council be appointed to decide as to the best method of securing the same."

### ST. HELENS PROVIDENCE FREE HOSPITAL.

This institution celebrated its jubilee last week, when Lord Derby laid the foundation stone of a new second ward for men, with more accommodation for women, and a children's hospital over it; and the Hon. Mary Lady Gerard, president of the Extension Committee, opened a new operating theatre and private wards; while, finally, the Duchess of Norfolk unveiled a memorial tablet to the founder of the hospital, Mother Magdalen Taylor, or Miss Taylor, as she is familiarly spoken of. The new wing now in course of erection provides accommodation for 16 beds, which number can be easily increased to 20 if ever required, in the men's ward, and similar accommodation for women and children above. The total cost of the new buildings is put down at £8,000, but a further £2,000 will be required for the equipment and furnishings. The extensions are being built to plans prepared by Mr. J. A. Baron, architect, St. Helens. Messrs. Westworth and Sons, St. Helens, have published an artistic souvenir of the hospital's jubilee, which gives plans and photographs of the institution.

### NATIONAL HOSPITAL FOR THE PARALYSED.

In connection with the jubilee of the National Hospital for the Paralysed and Epileptic (Albany Memorial), Queen Square, Bloomsbury, a thanksgiving service will be held in the hospital chapel on Tuesday, November 2, at 2.45 p.m., when an address will be given by the Archbishop of Canterbury. At 4 p.m. on the same day H.R.H. the Duchess of Albany will receive the Jubilee Purses.

### KENSINGTON AND FULHAM GENERAL HOSPITAL.

A special meeting of the governors was held at this hospital in Richmond Road, Earl's Court, on Saturday last, under the presidency of Lieutenant-Colonel H. J. F. Praegar, chairman of the board of management. The chairman, in opening the proceedings, explained that the liabilities of the institution amounted to £3,223, and were increasing. Colonel Gordon Young proposed that the hospital be placed unreservedly in the hands of the King Edward's Hospital Fund. Miss A. M. Thornett seconded the resolution, which was carried. As there was not a quorum of governors present, it is necessary that the decision shall be confirmed at another meeting.

### LEICESTER INFIRMARY.

The quarterly meeting of the Governors was held on the 20th inst., Sir Edward Wood, the Chairman of the Board, presiding over a very large gathering. The House Governor and Secretary (Mr. Harry Johnson) presented his quarterly report on the work of the institution, which showed that 1,960 adults had been admitted to September 30 to the wards of the Infirmary, as against 1,852 in the corresponding period of 1908, and 460 children had been admitted to the children's hospital, against 523 in the corresponding period. The daily number of beds occupied was 207.4 as compared with 186 in 1908. 9,644 new out-patients and 22,982 old out-patients had received treatment in the nine months, and 9,286 accident or casualty cases who had made 37,233 attendances; 829 *x*-ray cases had received attention in the *x*-ray department, in addition to a large number of "screen" cases. The records of operations showed 1,414 operations in the in-patients' department, 1,128 in the out-patients' department, 1,216 in the casualty department, making a total of 3,758 cases. The figures conveyed, as accurately as figures could, an idea of the enormous amount of sickness which the Infirmary and Children's Hospital were endeavouring to combat, and the governors would not fail to realise the care and devotion and the almost exacting nature of the duties of the hon. medical staff and not least the untiring energy of Miss Rogers, the Lady Superintendent, and her nursing staff. The income and expenditure was as follows :

| | Income to Sept. 30. | | Expenditure to Sept. 30. | |
|---|---|---|---|---|
| | 1908 | 1909 | 1908 | 1909 |
| Infirmary ... ... | 9,724 | 11,331 | 13,395 | 14,317 |
| Children's Hospital | 2,550 | 820 | | |

*Extension Fund.*—The new Nurses' Home (the last item of the reconstruction and enlargement scheme) would shortly be opened. Practically £100,000 would then have been spent, and, owing to the energies of the Chairman (whose success had caused the governors the liveliest satisfaction), only £5,250 remained unpaid, and this amount the Chairman was hopeful of reporting promised before the year closed. The House Governor, in conclusion, alluded to the visit of Sir Henry Burdett recently, and read a very gratifying report he had written in the distinguished visitors' book. Sir Edward Wood, in moving the adoption of the report, stated that the annual income must be increased by £3,000 if the 33 additional beds shortly to be opened were to be maintained without debt, and indicated the methods by which he hoped the money would be raised.

